# Long-term vertigo control after cochlear implantation in patients with end-stage Menière’s disease: A retrospective questionnaire-based cross-sectional study

**DOI:** 10.1007/s00508-019-01605-9

**Published:** 2020-01-29

**Authors:** Annabella Kurz, Alice Auinger, Christoph Arnoldner

**Affiliations:** Department of Otolaryngology, Head and Neck Surgery, Medical University of Vienna – AKH Vienna, Waehringer Guertel 18–20, 1090 Vienna, Austria

**Keywords:** Surgery, Endolymphatic hydrops, Deafness, Dizziness, Tinnitus

## Abstract

**Background:**

Menière’s disease (MD) is a symptom complex which is characterized by episodes of vertigo, tinnitus and fluctuating sensorineural hearing loss, which worsens during the course of the disease.

**Objective:**

Vertigo attacks (MD functional level scale) before compared to after cochlear implantation in patients with end-stage MD.

**Design and patients:**

In this questionnaire-based cross-sectional study eight patients with end-stage MD, who received a cochlear implant (CI) were analyzed.

**Main outcome measure:**

The effect of the CI on vertigo was measured preoperatively and postoperatively with the Menière’s disease functional level scale and the Menière’s disease outcome questionnaire. The primary outcome parameter influence of vertigo attacks on daily living was analyzed using the non-parametric Wilcoxon signed rank test before and after CI.

**Setting:**

Department of otolaryngology of a medical university.

**Results:**

The primary outcome measure influence of vertigo attacks on daily living as measured by the MD functional level scale improved significantly after CI.

**Conclusion:**

A CI can be an adequate treatment for vertigo attacks in patients with end-stage MD; however, due to the small sample size additional (multicenter) trials are necessary to confirm the findings.

## Introduction

Menière’s disease (MD) is a symptom complex which is characterized by episodes of vertigo, tinnitus, aural fullness and fluctuating sensorineural hearing loss, which worsen during the course of the disease [1].

This can lead to deafness on the affected side in many patients. Reported rates range from 5% to 50%. The incidence of bilateral deafness secondary to MD is rare and ranges from 1% to 6% [2].

Recommended MD treatment includes lifestyle modifications followed by administration of diuretics and betahistine, intratympanic corticosteroids, endolymphatic sac surgery, medical destructive treatment (intratympanic gentamicin treatment), and surgical destructive treatment (labyrinthectomy ± cochlear implantation, vestibular neurectomy) [3].

The impact of cochlear implantation (CI) on vertigo in MD is not yet fully understood. Whereas some studies reported a clear improvement after CI [4–6] others found little or no effect [6–8].

In MD, labyrinthectomy was first performed in 1895 as a destructive but very effective option for patients with treatment-resistant vertigo [9]. Recently this treatment has become more interesting again as hearing can be restored with a cochlear implant in a simultaneous procedure [10–13].

In 2013 Hansen et al. reported 10 end-stage unilateral MD patients who underwent labyrinthectomy and simultaneously CI, vertigo control was achieved in all of the cases [14]. This is not surprising as both modalities per se have been proven to be beneficial; however, the question whether cochlear implantation alone would improve vertigo spells in MD patients was not answered. In a retrospective study, Mukherjee et al. compared 22 MD patients (19 bilateral and 3 unilateral MD) who underwent CI only to 6 MD (5 unilateral and 1 bilateral) patients who received CI in combination with simultaneous labyrinthectomy and to 3 MD (2 unilateral and 1 bilateral) patients who received CI and delayed labyrinthectomy. Preoperatively in the CI only group two patients suffered from vestibular symptoms. Postoperatively long-lasting vestibular dysfunction occurred in three patients in the CI only group, whereas two of these patients presented with vestibular symptoms on the contralateral ear over many months. In the simultaneous CI group preoperatively six patients suffered from vertigo and stayed free of vertigo postoperatively [10]. Lustig et al. observed 9 CI MD patients, only 2 patients presented with vertigo attacks preoperatively, postoperatively only 1 patient suffered from vertigo in the first 3 months after implantation and stayed vertigo-free up to 4.5 years [4].

In summary, little is known about the impact of CI on vertigo in patients suffering from recalcitrant MD. For that reason, the primary aim was to evaluate if CI improves vertigo and secondary if CI improves the quality of life in patients with end-stage MD.

## Patients, materials and methods

### Primary outcome

This questionnaire-based cross-sectional study was performed at the department of otolaryngology, head and neck surgery of a university hospital and was approved by the local ethics committee. A total of eight patients suffering from MD according to the AAO guidelines and who received a cochlear implant (MED-EL [Elektromedizinische Geräte Gesellschaft m. b. H., Innsbruck, Austria]) due to severe sensorineural hearing loss were included in the study. The surgery was performed between June 2002 and July 2013. All subjects were selected based on information from their patient charts (Table 1). All subjects were deaf on the MD ear preoperatively. Patients with bilateral MD received only unilateral CI, which was implanted on the side with the more pronounced hearing loss. Subjects were requested to answer several questionnaires from a preoperative and a present perspective at follow-up time. The questionnaires included the morbus Menière outcome questionnaire and the MD functional level scale [15]. The primary goal was to evaluate the effect of a CI on vertigo spells in patients with end-stage MD according to the functional level scale before compared to after CI. Secondary outcome measures were quantity of vertigo attacks (*n* per month) perioperatively (time period of 6 months before vs. 6 months after CI) and quality of life measured by the morbus Menière outcome questionnaire.

**Table 1:** Patient characteristics

Pat	Age at implantation (years)	Sex	Bilateral MD (y/n)	Cochlear implant	Duration of deafness (years)	Time from CI to follow-up (years)	Follow-up date	Treatment prior to CI
1	78.3	M	*n*	MED-EL SONATA STANDARD	20.2	6.8	04.2018	Conservative
2	63.2	M	y	MED-EL COMBI 40+ STANDARD	28.5	13.6	05.2016	Conservative
3	48.7	M	*n*	MED-EL SYNCHRONY FLEX 28	20.8	4.2	07.2016	Conservative
4	67.9	F	y	MED-EL CONCERTO FLEX SOFT	24.8	5.3	04.2018	Conservative
5	70.5	F	y	MED-EL CONCERTO FLEX 28	N/A	3.0	05.2016	Tenotomy
6	67.6	F	y	MED-EL CONCERTO FLEX 28	5.3	4.7	04.2018	Conservative
7	70.3	M	*n*	MED-EL PULSAR FLEX 28	N/A	5.6	05.2016	Conservative
8	50.8	M	*n*	MED-EL CONCERTO STANDARD	18.5	2.5	06.2016	Gentamicin injection
Mean	64.7	–	–	–	19.7	5.7	–	–

*MD* Menière’s disease, *CI* cochlear implant

### Statistical analysis

Depending on distributional properties continuous data were expressed as medians (25th–75th percentile). Binary data were expressed as counts and relative frequencies. Changes in MD symptoms were assessed by the non-parametric Wilcoxon signed rank test. Multivariate regression analysis was used to assess influence of age, sex and time from diagnosis of MD to CI on the outcome parameter difference of number of vertigo attacks before vs. after CI. For data management and statistical analyses Microsoft Excel 2010 (Microsoft Corporation, Redmond, WA, USA) and IBM SPSS (version 21, IBM, Armonk, NY, USA) for Windows was used. A *p*-value of < 0.05 was regarded to indicate statistical significance. Sample size calculation was not performed since this is a pilot study and no meaningful was available at that moment.

The study was approved by the local Ethics Committee. All procedures followed were in accordance with the ethical standards of the responsible committee on human experimentation (institutional and national) and with the Helsinki Declaration of 1975, as revised in 2008.

### Questionnaires

The Menière’s disease functional level scale reflects the influence of MD in patients daily living activities on a scale from 1 to 6 [15].

### Menière’s disease outcome questionnaire

This questionnaire consists of 40 questions which records the quality of life before and after a treatment for MD. A special emphasis is on the activities of daily living. A lower score yields a higher quality of life [16].

## Results

This analysis included eight patients suffering from end-stage MD who received a CI (Table 1), four with unilateral MD and four with bilateral MD. Follow-up time after implantation ranged from 2.5–13.5 years (mean 6.7 years).

The primary outcome measure influence of vertigo attacks on daily living as measured by MD functional level scale improved significantly after CI (before vs. after CI: 5 (4–5) vs. 3 (2–4); *p* = 0.027; Table 2). The secondary outcome quantity of vertigo attacks per month perioperatively decreased insignificantly after CI (before vs. after CI: 2 (1–4) vs. 1 (0–1); *p* = 0.058), while the quality of life as measured by the Menière’s disease outcome questionnaire improved significantly after CI (before vs. after CI: 45 (34–48) vs. 27 (17–35); *p* = 0.035; Table 2).

**Table 2:** Primary and secondary outcome variables

	Before CI	After CI	*p*-value
Vertigo attacks (Menière’s disease functional level scale)	5 (4–5)	3 (2–4)	0.027
Quantity of vertigo attacks (*n* per month) perioperatively ^a^	2 (1–4)	1 (0–1)	0.058
Quality of life (Menière’s disease outcome questionnaire)	45 (34–48)	27 (17–35)	0.035

Data are median (25th–75th percentile), unless otherwise stated

*CI* cochlear implant

^a^ 6‑ month time period before CI vs. 6‑month time period after CI

In multivariate regression analysis no covariables were significantly predictive for the outcome parameter difference of number of vertigo attacks before vs. after CI.

## Discussion

The impact of CI on vertigo in patients with MD is not yet fully understood; however, based on the assumed pathophysiology, patients may benefit from implantation as the inner ear is opened via the round window or cochleostomy and consequently pressure is relieved. Overall, some patients have only few vertigo attacks preoperatively reflecting the end-stage disease; others show no attacks due to previous surgery to control vertigo and some others still have no vertigo attacks even in the end-stage of MD [17].

In this study, in eight patients with end-stage MD it could be shown that CI improved vertigo and quality of life with follow-up times of 3–14 years.

The results are consistent with findings from McRackan et al. who examined 21 MD patients (unilateral or bilateral disease) after CI. Of these patients six suffered from vertiginous symptoms preoperatively. Postoperatively two patients were vertigo-free, three reported improvements and one had constant vertigo attacks [5]. Mick et al. investigated 20 patients with either unilateral or bilateral MD: 6 subjects suffered from at least 1 episode of vertigo in the year before surgery. Postoperatively, one patient continued to suffer from lightheadedness, which resolved spontaneously, five other patients reported chronic dizziness after implantation; however, these five patients had bilateral MD and were implanted only on one side [6].

It needs to be stated that in the abovementioned studies not all patients with MD were implanted on the affected side compared to the present study.

Fife et al. found no vertigo attacks postoperatively in 10 CI MD patients with a follow-up of 6 years amd 4 of these patients underwent surgical or chemical labyrinthectomy preoperatively [7].

Lustig et al. investigated seven patients with bilateral MD and two with unilateral MD after CI. Of these patients five had undergone previous surgery for vertigo control, e.g. sac decompression, shunting, perilymphatic fistula exploration and labyrinthectomy. Interestingly, only the labyrinthectomized patient suffered from vertigo in the first month of implantation [4]. Hence, vertigo control rates were clearly higher in the present study, where only one patient had a previous surgery for vertigo (tenotomy) (Table 1), another patient had received gentamicin injections.

The combination of labyrinthectomy and CI has been recently described as a successful tool of vertigo control and restoration of hearing [10–14]; however, after labyrinthectomy, postoperative vertigo can occur due to inadequate surgical removal of the vestibular sense organs, neuroma formation in the vestibule, and high regenerative potential of the vestibular nerve [18, 19].

In a study Doobe et al. reported 5 unilateral MD patients undergoing labyrinthectomy and CI with vertigo relief lasting more than 6 months [11]. Osborn et al. reported delayed CI 21 years after labyrinthectomy in a case report in a bilateral MD patient [20]. In contrast Hansen et al. performed simultaneous labyrinthectomy and CI in 10 unilateral MD patients to avoid a second anesthesia and ossification in the cochlea resulting in problems during implantation and reduction of deafness time. In all 10 unilateral MD patients vertigo control was achieved, 4 with a follow-up time less than 6 months and a few patients with a follow-up time 18–24 months [14]. Although labyrinthectomy is an adequate tool for vertigo relief, it is a destructive surgery and associated with a risk of cochlear ossification and impaired CI performance. In the present study, vertigo control was achieved with CI only. As a second step, labyrinthectomy could still be performed in patients with persistent vertigo attacks after CI implantation.

### Limitations of the study

The small sample size and the retrospective character of this trial is a significant limitation.

## Conclusion

In end-stage MD patients CI may control vertigo; however, due to the small sample size additional prospective (multicenter) trials are necessary to confirm these findings.
